# Supplementary figures and images for: Further Mining and Characterization of miRNA Resource in Chinese Fir (Cunninghamia lanceolata)
Source: Genes (Basel). 2022 Nov 17;13(11):2137. doi: 10.3390/genes13112137 (PMC9690001; doi:10.3390/genes13112137)

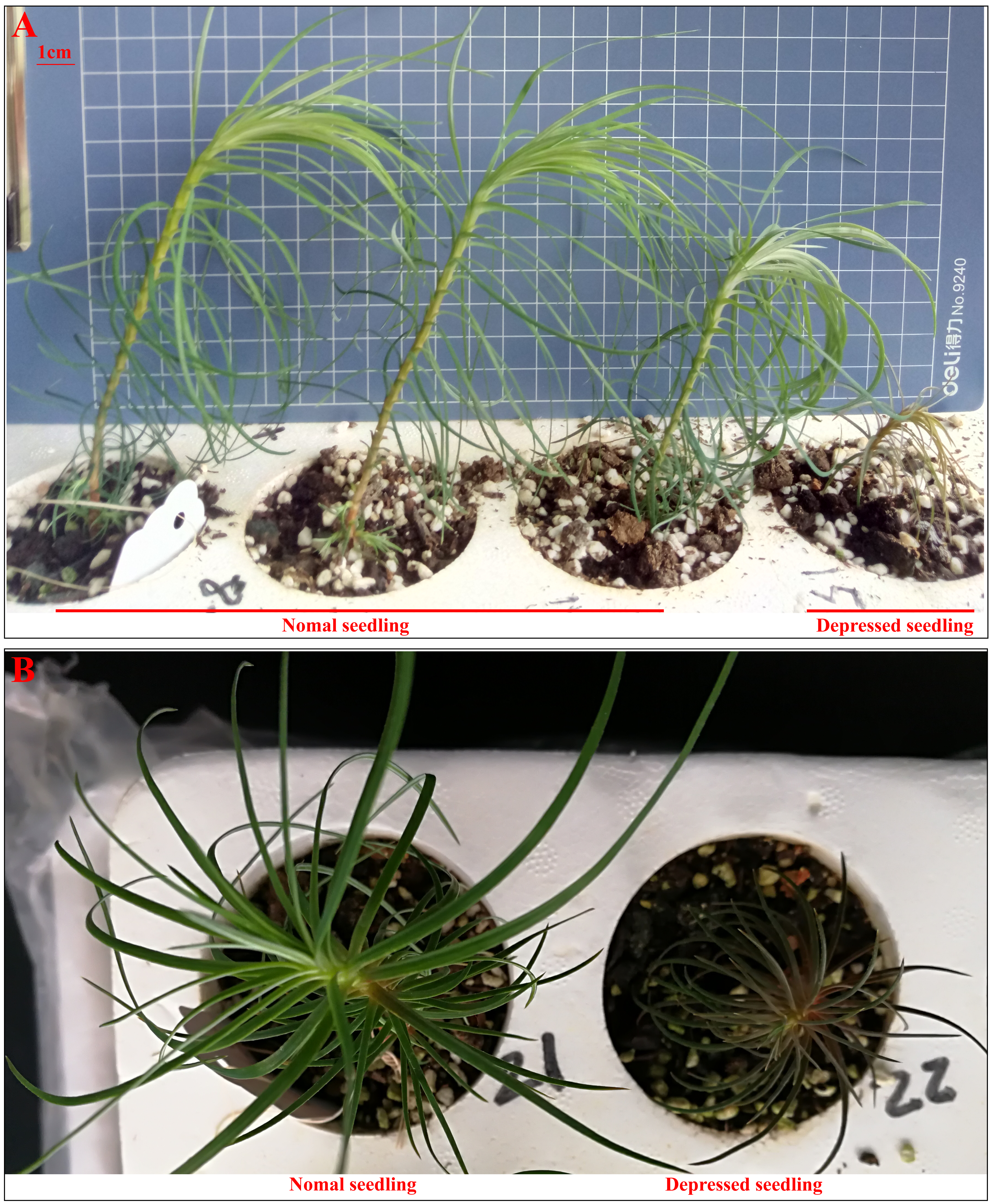

Supplement: Supplementary file 1 [file genes-13-02137-s001.zip › Supplemental Figure S1 The contrast photos of the morphological features in selfed progeny of Chinese fir clone cx569.jpg]

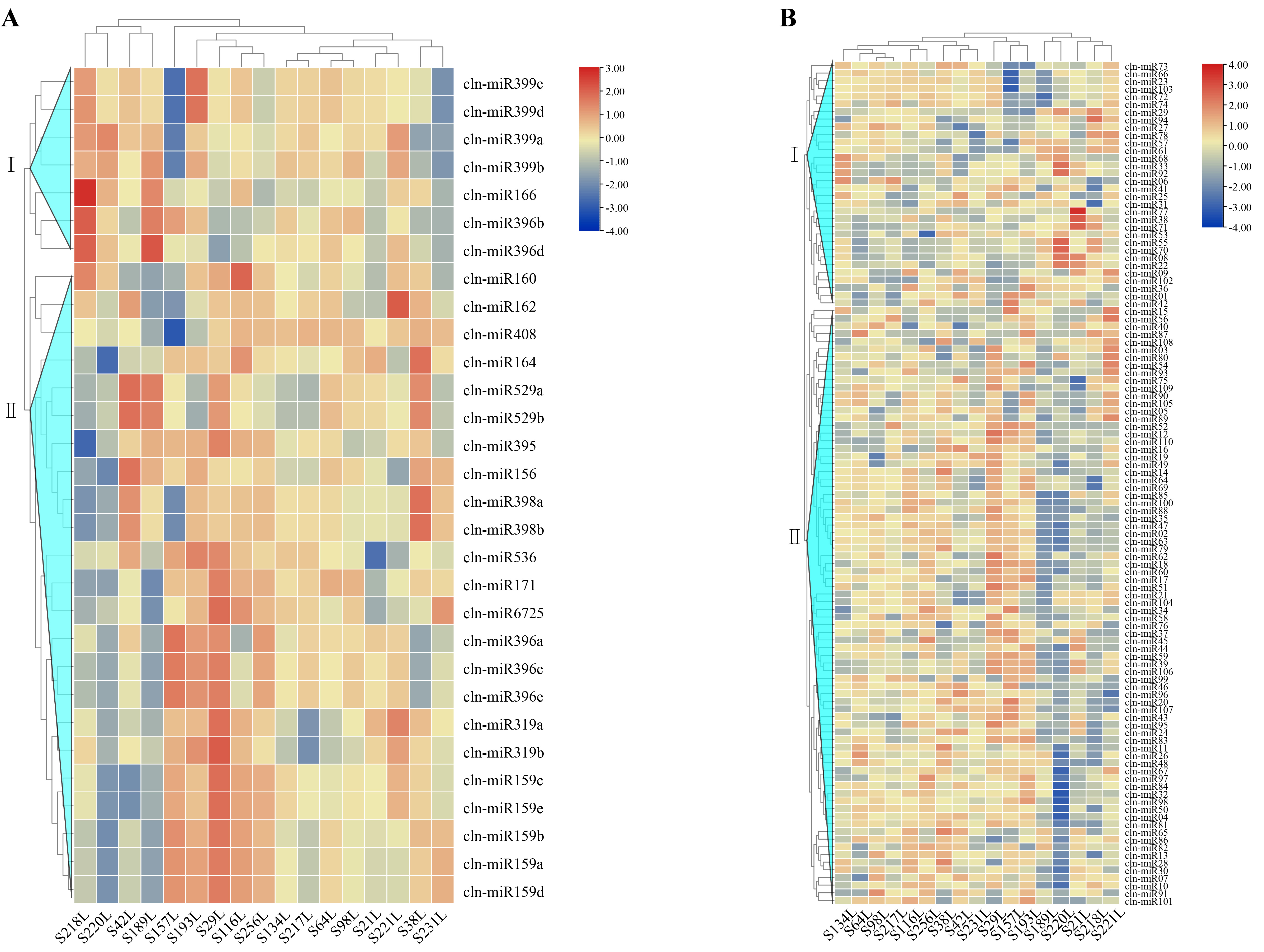

Supplement: Supplementary file 1 [file genes-13-02137-s001.zip › Supplemental Figure S2 Transcript profiles of known and novel miRNAs.jpg]

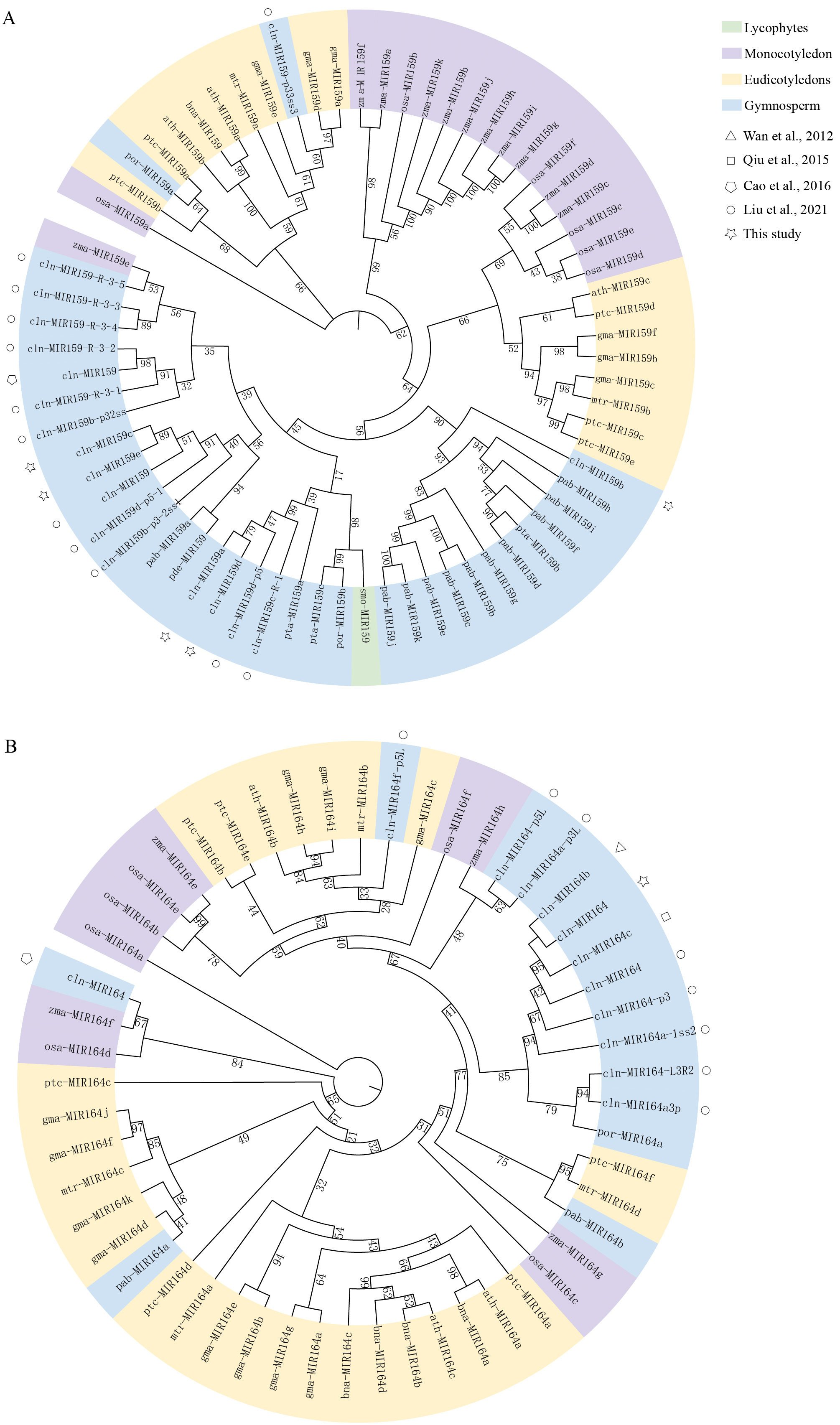

Supplement: Supplementary file 1 [file genes-13-02137-s001.zip › Supplemental Figure S3 Phylogenetic analysis of pre-miR159 and pre-miR164 between Chinese fir and other plant species.jpg]

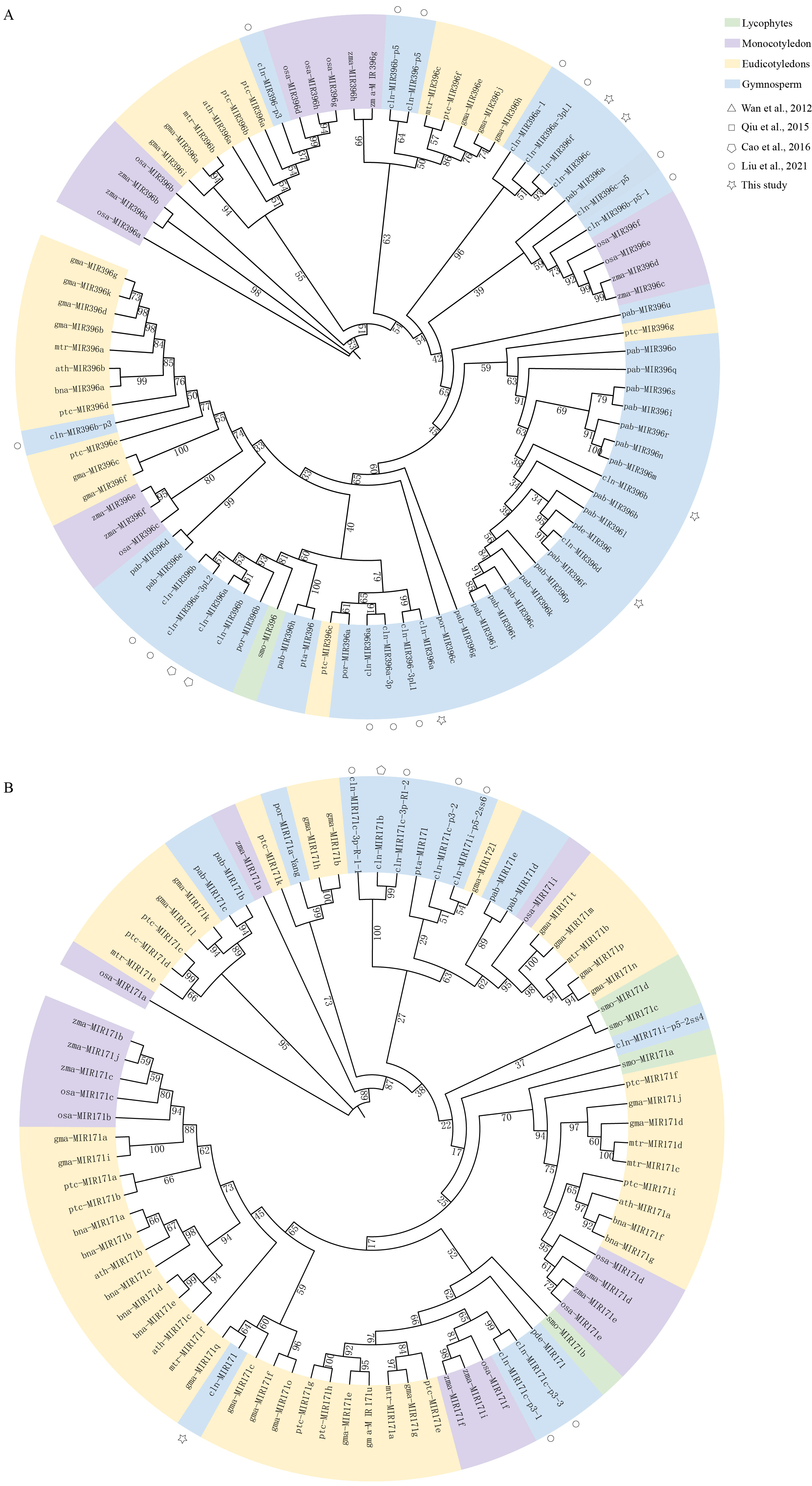

Supplement: Supplementary file 1 [file genes-13-02137-s001.zip › Supplemental Figure S4 Phylogenetic analysis of pre-miR396 and pre-miR171_1 between Chinese fir and other plant species.jpg]
